# Large molecules from the cerebrospinal fluid enter the optic nerve but not the retina of mice

**DOI:** 10.1186/s12987-023-00506-4

**Published:** 2024-01-04

**Authors:** Xiao J. Tong, Gokhan Akdemir, Meetu Wadhwa, Alan S. Verkman, Alex J. Smith

**Affiliations:** 1 Department of Ophthalmology, University of California San Francisco, San Francisco, CA 94131 USA; 2 Departments of Medicine and Physiology, University of California San Francisco, San Francisco, CA 94131 USA

**Keywords:** Aquaporin-4, Glymphatics, Neuromyelitis optica spectrum disorder

## Abstract

It has been proposed that cerebrospinal fluid (CSF) can enter and leave the retina and optic nerve along perivascular spaces surrounding the central retinal vessels as part of an aquaporin-4 (AQP4) dependent ocular ‘glymphatic’ system. Here, we injected fluorescent dextrans and antibodies into the CSF of mice at the cisterna magna and measured their distribution in the optic nerve and retina. We found that uptake of dextrans in the perivascular spaces and parenchyma of the optic nerve is highly sensitive to the cisternal injection rate, where high injection rates, in which dextran disperses fully in the sub-arachnoid space, led to uptake along the full length of the optic nerve. Accumulation of dextrans in the optic nerve did not differ significantly in wild-type and AQP4 knockout mice. Dextrans did not enter the retina, even when intracranial pressure was greatly increased over intraocular pressure. However, elevation of intraocular pressure reduced accumulation of fluorescent dextrans in the optic nerve head, and intravitreally injected dextrans left the retina via perivascular spaces surrounding the central retinal vessels. Human IgG distributed throughout the perivascular and parenchymal areas of the optic nerve to a similar extent as dextran following cisternal injection. However, uptake of a cisternally injected AQP4-IgG antibody, derived from a seropositive neuromyelitis optica spectrum disorder subject, was limited by AQP4 binding. We conclude that large molecules injected in the CSF can accumulate along the length of the optic nerve if they are fully dispersed in the optic nerve sub-arachnoid space but that they do not enter the retina.

## Introduction

Perivascular spaces surrounding penetrating arterioles connect the sub-arachnoid space to the parenchyma of CNS tissues and facilitate exchange of solutes between the interstitial fluid (ISF) and cerebrospinal fluid (CSF) [1]. In the brain, parenchymal uptake of dextrans, antibodies and proteins injected into the CSF at the cisterna magna is controlled by factors including the rate of clearance from the sub-arachnoid space, the permeability of the pial membrane, and the size-dependent rate of diffusion within the parenchyma [2–4]. Dextrans injected into the CSF also reach the parenchymal and perivascular areas of the optic nerve, due to continuity of the optic nerve sub-arachnoid space with that surrounding the brain [5]. It has further been proposed that CSF solutes can circulate through the retina via perivascular spaces surrounding the central retinal artery as part of an AQP4-mediated ocular glymphatic system [6]. Previous studies have demonstrated that macromolecules injected into the vitreous of rodents can drain along the perivascular spaces surrounding the central retinal vessels [7, 8]. However, it remains unclear if CSF macromolecules can enter the retina by this pathway, given the well-characterized pathways for CSF drainage in the periorbital tissues and sclera [9–11].

The ocular glymphatic hypothesis, like the glymphatic hypothesis in the brain, proposes that glial water transport via AQP4 is a key regulator of the transport of macromolecular solutes between the subarachnoid space and interstitium [12]. The role of AQP4 in this process in brain remains controversial [13]. Additionally, AQP4 is not expressed in astrocytes of the optic nerve head [14] and is therefore unlikely to be involved in regulating perivascular transport along the central retinal vessels. Determining if AQP4 regulates transport of solutes from the CSF to optic nerve is of particular importance in AQP4-IgG seropositive neuromyelitis optica spectrum disorder (NMOSD) where autoantibodies to AQP4 cause loss of AQP4 from optic nerve astrocytes and optic neuritis [14, 15].

Clearance of intracisternally injected macromolecular solutes from the sub-arachnoid space to CSF-draining lymphatics limits the amount of solute that can enter the perivascular spaces of the brain [3]. Cisternal injection protocols that disrupt endogenous CSF movement and disperse macromolecules within the CSF before they are cleared can therefore greatly increase tissue uptake of these solutes [16]. The consequences of dural clearance for optic nerve and retinal tracer uptake remain unclear but are likely to be important given optic nerve structure and the high concentration of lymphatics around the optic nerve head. Building on our previous work demonstrating the sensitivity of tracer uptake in brain to injection conditions [16], we have investigated fluid movement between CSF, optic nerve and retina.

## Methods

*Animals.* Experiments were performed on wild-type and AQP4 deficient mice on the c57 Bl/6 background strain [17] at 2–4 months of age. All animal procedures were approved by the University of California, San Francisco Institutional Animal Care and Use Committee.

*Materials*. Fixable fluorescent dextrans (FITC, 2,000 kDa; TRITC, 70 kDa; Alexa Fluor 647, 10 kDa; Molecular Probes) were dissolved at 10 mg/ml in artificial CSF (aCSF), stored as frozen aliquots at − 70 °C, and diluted with aCSF to specified concentrations before use. Antibodies used were rat monoclonal anti-CD31 antibody (550,274; BD PharMingen), rabbit polyclonal anti AQP4 antibody, recombinant human AQP4-IgG clone r53 [18] and purified human immunoglobulin (IVIg). Alexa fluor-labeled secondary antibodies were from Molecular Probes. Other chemicals were from Sigma-Aldrich.

*Intracisternal injection*. Intracisternal injection was done as previously described [16]. Mice were anesthetized with ketamine (100 mg/kg) and xylazine (10 mg/kg) and immobilized on a stereotaxic frame, and the dura overlying the cisterna magna was surgically exposed. A 30-g needle attached to a syringe pump via polyethylene tubing, which was preloaded with the specified volume and concentration of solutes, was inserted into the cisterna magna and cemented in place with cyanoacrylate glue. At the end of injections, the tubing was heat cauterized to prevent backflow, and the needle was left in place. Mice remained immobilized on the stereotaxic frame for 25 min. after injection and were then removed from the frame and transported to a fume hood for transcardial perfusion fixation. Perfusion was initially with cold, heparinized PBS for 3 min, and then with 4% paraformaldehyde in PBS for a further 12 min. The total interval between the removal of mice from the stereotaxic frame to the arrival of fixative in the brain was estimated at 5 min. After perfusion, the skull was placed in 4% paraformaldehyde in PBS prior to optic nerve removal.

*Intraocular pressure manipulation.* A beveled glass pipet with tip diameter of 20–30 μm was connected to a fluid column of fixed height. The pipet tip was then inserted in the anterior chamber through the cornea using a micromanipulator and the connection to the fluid column was opened. Intraocular pressure was recorded with a handheld tonometer at 1 min. intervals. For reduction of IOP, a 30G needle was used to puncture the anterior chamber through the cornea and then removed to allow the fluid to drain.

*Intravitreal injection.* Mice were anesthetized with ketamine (100 mg/kg) and xylazine (10 mg/kg) and immobilized on a stereotaxic frame. 1 µl of a solution containing 10 kDa, 70 kDa and 2000 kDa fluorescent dextrans at a concentration of 2.5 mg/ml in aCSF was injected intravitreally using a 32G Hamilton syringe. Animals were euthanized by transcardial perfusion with 4% paraformaldehyde 4 h after intravitreal injection and the eye was removed and sectioned for imaging by confocal microscopy.

*Sample preparation and imaging.* In experiments to determine uptake of CSF-injected dextrans in the retrobulbar optic nerve, the fixed optic nerve was cut at the point it enters the eye, mounted on microscope slides in ProLong Gold antifade (Molecular Probes) and imaged by confocal microscopy. In experiments to compare distribution of cisternally injected macromolecules with immunolabelled molecules in the optic nerve, samples were cryopreserved after fixation and mounted for transverse sectioning through the optic nerve. For costaining with vascular markers, 20-µm-thick frozen sections were cut and stained with the antibodies and mounted on slides for imaging by confocal microscopy. Images were captured on a Zeiss LSM700 confocal microscope using 10x/0.3 NA, 20x/0.8NA or 63x/1.4NA objectives.

*Image analysis.* Measurement of tracer intensity within specific image ROIs was done with FIJI software. Sub-arachnoid space fluorescence intensity (F^S^), perivascular fluorescence intensity (F^PV^) and parenchymal fluorescence intensity (F^par^) were determined within manually defined ROIs based on the characteristic anatomy of each compartment. Graphing and statistical analysis were done with GraphPad Prism software.

## Results

### Solute transport from CSF to optic nerve is dependent on CSF injection protocol

**Fig. 1:**
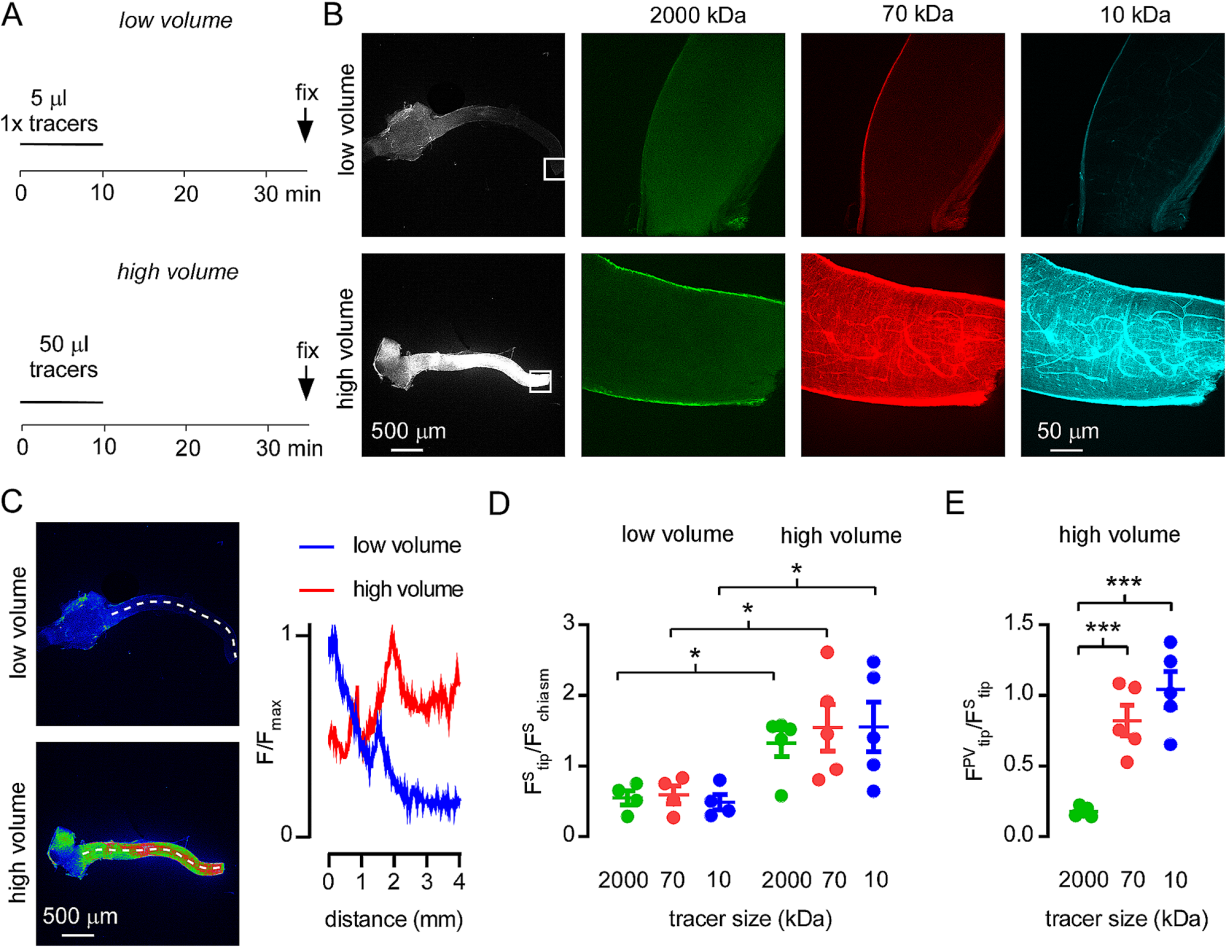
Uptake of cisternally-injected dextrans along the optic nerve requires high volume injection. (**A**) Injection protocol for dispersion of dextrans in the sub-arachnoid space. A fixed quantity of fluorescent lysine-dextrans (FITC-2000 kDa, TRITC 70 kDa and Alexa 647 10 kDa) were injected at either low (0.5 ul/min) or high (5 ul/min) injection rates for 10 min. Animals were sacrificed by perfusion fixation 30 min after the termination of injections and the optic nerve was removed for visualization by confocal microscopy. (**B**) *Left panel* Low magnification images showing fluorescence of 70 kDa TRITC-dextran in the dissected optic nerve from animals injected according to the protocols described in A. *Center and* r*ight panels* High resolution images of the boxed regions at left, corresponding to regions adjacent to the point where the optic nerve enters the eye, showing uptake of each of the fluorescent dextrans. (**C**) *Left* Images of the optic nerve from B, pseudocolored to show fluorescence intensity, where red indicates high intensity, blue indicates low intensity and green indicates intermediate intensity. The dotted lines were used to calculate intensity profile. *Right.* Relative fluorescence intensity as a function of distance from the optic chiasm for optic nerves with high and low volume injection. (**D**) Quantification of the ratio of sub-arachnoid fluorescence intensity between chiasmal and tip regions of the optic nerve for each tracer under high or low volume injection conditions (n = 4 low volume vs. 5 high volume. (*p < 0.05 between high and low volume injection by t-test). (**E**) Analysis of the ratio between perivascular tracer intensity and sub-arachnoid intensity for each tracer after high volume injection (n = 5 ***p < 0.001 by t-test))

Fluid movements between the CSF, optic nerve and retina are an important component of numerous ocular diseases. We have previously shown that brain uptake of solutes injected in the CSF is highly sensitive to injection conditions, where large volume injections increase solute dispersal in the CSF and greatly increase tissue penetration [16]. To determine if similar mechanisms determine the extent to which CSF solutes enter the optic nerve, we injected fixable, fluorescent dextrans of 10, 70 and 2000 kDa molecular size into the CSF at the cisterna magna and determined their relative fluorescence intensity along the optic nerve, from the optic chiasm to the optic nerve head. A fixed amount of dextran was delivered via a large bore injection needle in either a small volume (5 µl at 0.5 µl/min), which minimally disrupts endogenous CSF movement, or in a large volume (50 µl at 5 µl/min), which rapidly disperses injected molecules throughout the CSF (13; Fig. 1A). Mice were then perfusion fixed 30 min. after injection, and the whole optic nerve was removed and imaged by confocal microscopy (Fig. 1B). With low volume injections, a gradient of dextran fluorescence with distance from the optic chiasm was observed, whereas with high volume injection, dextran uptake was similar along the length of the optic nerve (Fig. 1C). This finding was confirmed in analyses of dextran fluorescence from multiple experiments, where similar dextran uptake was seen throughout the optic nerve following high volume injection but uptake was confined to regions around the optic chiasm following low volume injection (Fig. 1D).

In cortical regions of the brain, transfer of dextrans from CSF to the perivascular spaces is size-independent for molecules as large as 2000 kDa but solute transfer into the parenchyma is diffusive and size dependent [19]. In the optic nerve, all three dextrans were present in the sub-arachnoid space following high volume injection (Fig. 1B, D), but only 10 and 70 kDa dextran entered the perivascular spaces of the nerve (Fig. 1E). The difference in perivascular uptake of 2000 kDa dextran between brain and optic nerve suggests regional variation in pial permeability to different sized solutes.

### Intracisternally injected dextrans do not accumulate in the retina

**Fig. 2:**
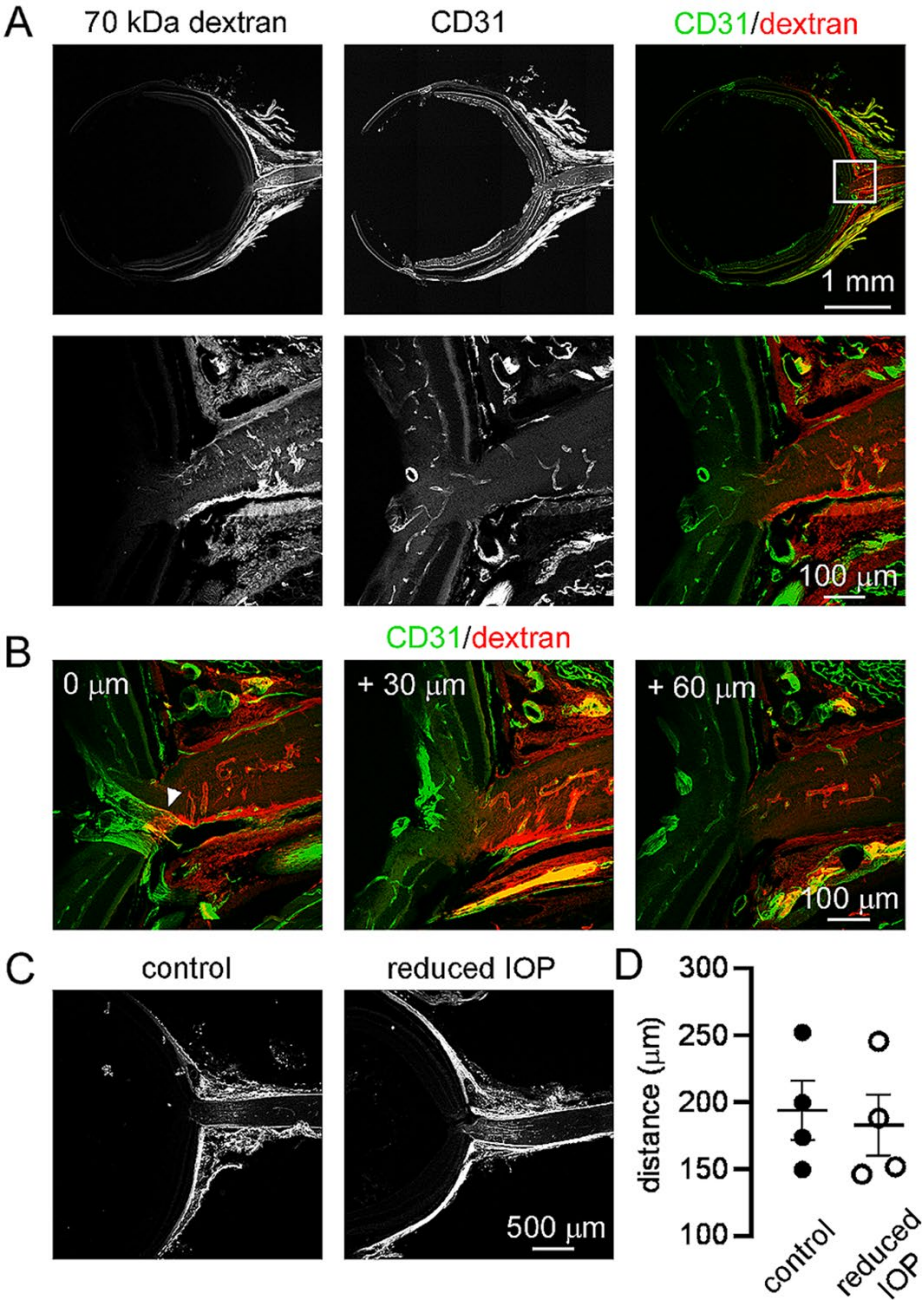
Cisternally injected solutes accumulate in the optic nerve head and sclera but not in the retina (**A**) Transverse section cut through the eye of a mouse after high volume cisternal injection of 70 kDa fixable TRITC-dextran following the protocol used in Fig. 1. Sections were subsequently stained with antibodies to CD31 (vascular endothelium). (**B**) Serial sections at 30 μm intervals through the optic nerve head from an animal injected and stained as in A. The arrowhead indicates cisternally injected dextran in the perivascular space surrounding the central retinal vessels in the optic nerve head but not further into the retina. (**C**) Distribution of 70 kDa tracer in a control eye (top) and in the contralateral eye (bottom) where intraocular pressure had been reduced by draining the anterior chamber. (**D**) Measurement of the minimum distance of visible dextran fluorescence from the retinal surface in control eyes and eyes with reduced IOP. No significant difference was observed

Having identified cisternal injection conditions which lead to uptake of dextrans along the optic nerve, we next determined if CSF solutes can enter the retina using the high-volume injection protocol shown in Fig. 1A. After dextran injection, mice were perfusion fixed, the eye and optic nerve was removed, and sections were cut through the optic nerve head then stained with antibodies to CD31 to mark blood vessels. Confocal images demonstrated that dextrans did not enter the optic nerve, but instead accumulated in the sclera and connective tissues surrounding the optic nerve head (Fig. 2A). In some samples, the central retinal vessels could be tracked across several sections as they entered the retina (Fig. 2B). Observations of cisternally injected dextran distribution in these samples revealed that dextrans could enter the perivascular space surrounding the central retinal vessels but did not progress further than the optic nerve head.

Intraocular pressure (IOP) is usually higher than intracerebral pressure (ICP), however the high volume cisternal injections used here elevate intracranial pressure by approximately 8 mmHg [16], to levels higher than the IOP commonly observed in ketamine/xylazine anesthetized animals [20]. To determine if further reducing the IOP allowed CSF dextran to enter the retina, we deflated one eye by draining the anterior chamber before cisternal dextran injection. This procedure reduced the IOP from a baseline average of 9.7 mmHg to below a level that could accurately be measured with a handheld tonometer. The extent to which cisternally injected dextrans penetrate the retina was then compared between deflated eyes and the contralateral control eye (Fig. 2C, D). No changes in the retinal penetration of tracer between eyes was observed in this experiment, demonstrating that an adverse pressure gradient is not the reason that dextrans are not observed in the retina.

**Fig. 3:**
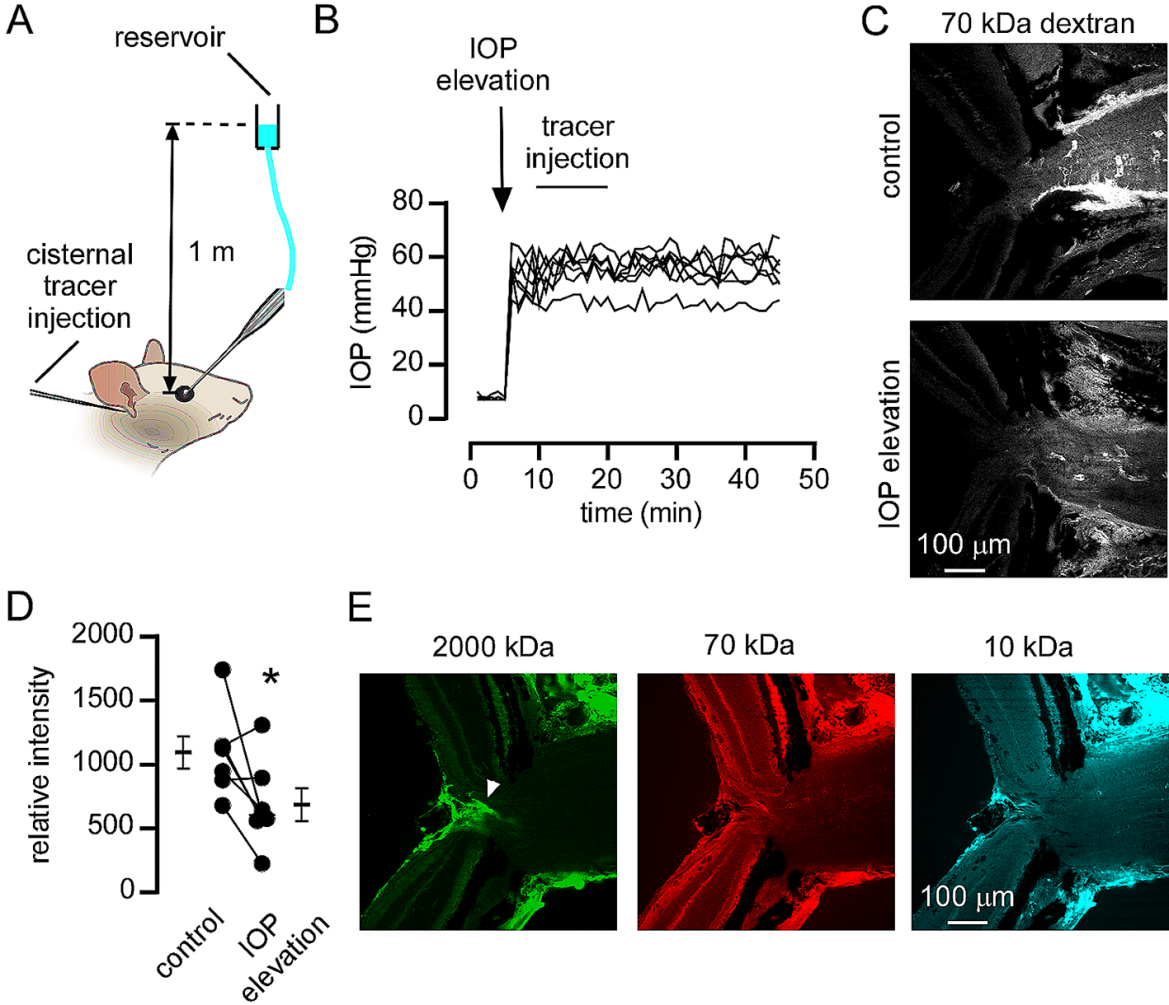
Fluid flow from the retina to the optic nerve via the perivascular spaces of the central retinal vessels (**A**) Experimental set up for determining the effect of elevated IOP on accumulation of cisternally injected dextrans in the optic nerve. (**B**) Measurements of intraocular pressure in eyes where pressure was acutely raised by anterior chamber cannulation (arrow) before and during cisternal injection of dextrans. Pressure measurements from 7 separate experiments are shown. (**C**) 70 kDa dextran tracer accumulation in the optic nerve head in a control eye or in the contralateral eye where IOP was elevated. (**D**) Relative fluorescent intensity of dextran tracer in control eyes and eyes where IOP was elevated (*p < 0.05 by paired t-test, n = 7). (**E**) Distribution of 3 different dextrans in the optic nerve head after intravitreal injection. The indicated dextrans were co-injected intravitreally and fixed by perfusion fixation 6 h later. Distribution of the dextrans in a slice through the optic nerve head is shown, the arrowhead indicates dextran accumulation around the central retinal vessels

Pressure-dependent transport of fluid from the vitreous into the optic nerve head has been previously reported [8] and chronic elevation of intraocular pressure has been shown to reduce accumulation of CSF solutes in the optic nerve [21]. Here we used an acute IOP elevation protocol [22] to determine if CSF movement into the optic nerve head was sensitive to IOP. IOP was elevated by inserting a glass pipet attached to a 1 m PBS filled column into the anterior chamber of the eye (Fig. 3A). IOP was immediately elevated to 40–60 mmHg by this procedure and remained constant at this level during cisternal dextran injection (Fig. 3B). The distribution of dextran in the optic nerve head was similar between eyes where IOP was elevated and control, contralateral eyes where IOP was not changed (Fig. 3C). However, the overall intensity of dextran fluorescence was significantly reduced in the optic nerve head of eyes with elevated IOP (Fig. 3D), consistent with the hypothesis that IOP elevation disrupts fluid transport in the optic nerve head. To further confirm that fluid can drain from the eye via the optic nerve head, we intravitreally injected a mix of differently sized, fixable, fluorescent dextrans and determined their distribution in the optic nerve head from eyes fixed 6 h later. These experiments confirmed that clearance of fluid from the eye into the optic nerve head via the central retinal vessels (Fig. 3E). Interestingly, even large (2000 kDa) solutes could enter the perivascular space surrounding the central retinal vessels, further demonstrating heterogeneity in the size-selectivity of perivascular macromolecule uptake. These results demonstrate that CSF does not enter the retina under the conditions of our experiment but that fluid transport from the retina to the optic nerve can occur.

### Solute transfer from CSF to optic nerve is not dependent on Aqp4

**Fig. 4:**
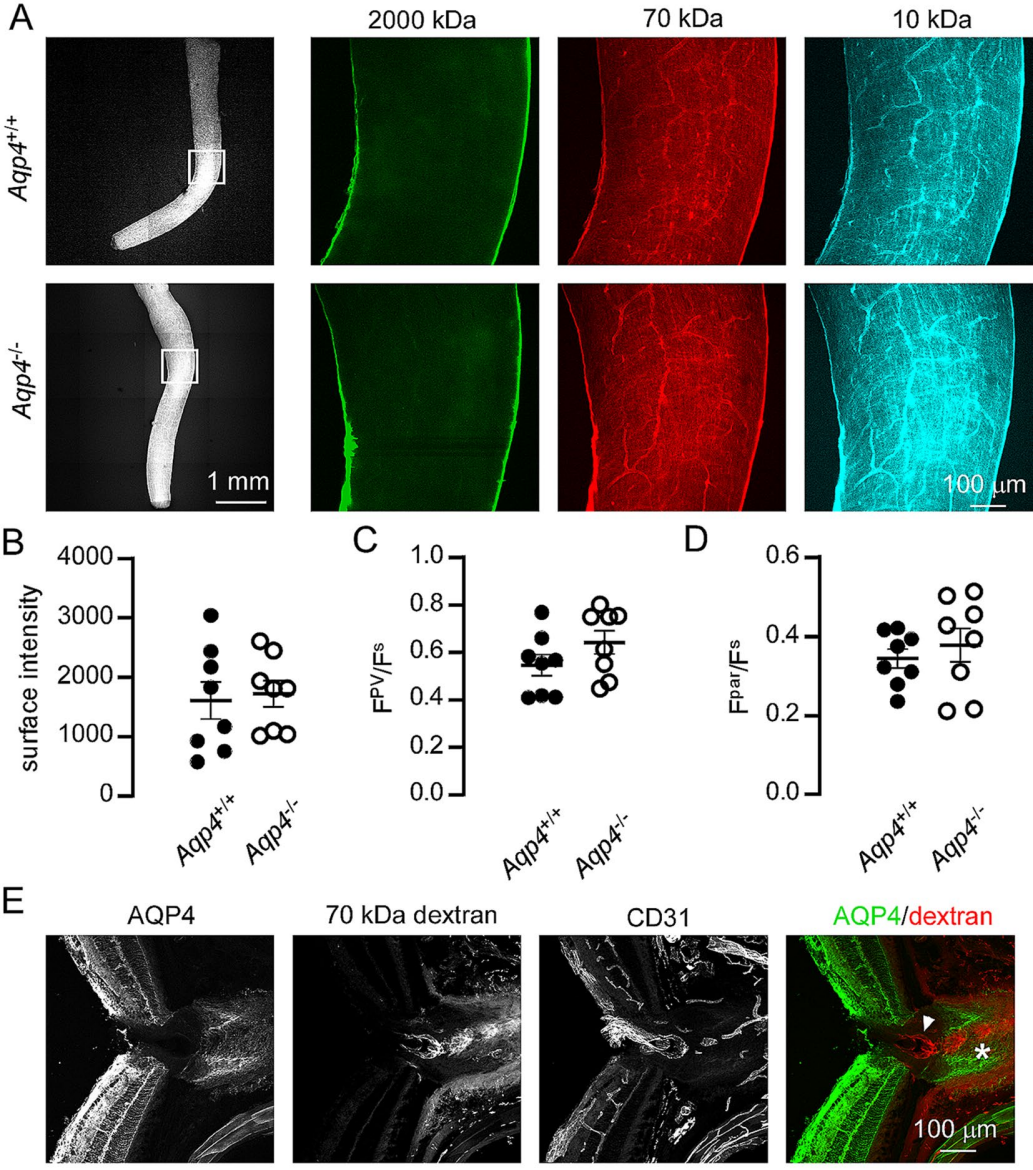
AQP4 is not required for uptake of cisternally-injected dextrans by the optic nerve. (**A**) *Left* Low magnification image showing fluorescence of 70 kDa dextran in acutely dissected optic nerves following high volume injection into the cisterna magna. *Center and right panels.* High magnification images showing distribution of co-injected 2000 kDa, 70 kDa and 10 kDa dextrans within the boxed regions shown at left. (**B–D**) Quantification of 70 kDa dextran intensity in the optic nerve sub-arachnoid space’ perivascular space and parenchyma from individual animals of each genotype (no significant difference in mean intensity was observed between genotypes by t-test (n = 8 each). (**E**) Tracer uptake in AQP4 expressing and non-expressing regions of the optic nerve. Frozen sections of the optic nerve head were prepared following high-volume injection with 70 kDa fluorescent dextran, then stained with antibodies to AQP4 and CD31. Tracer was observed in perivascular spaces of both regions that do (asterisk) and do not (arrowhead) express AQP4

The ocular glymphatic hypothesis posits that AQP4 is required for fluid exchange between CSF and ocular tissues [6, 8]. To determine if AQP4 is required for optic nerve uptake of solutes from CSF, we measured the intensity of cisternally injected fluorescent dextrans in the optic nerve from wild-type and AQP4 deficient mice (Fig. 4A). Averaged results from multiple animals showed no significant difference in dextran fluorescence intensity between genotypes (Fig. 4B–D). Uniquely in the murine CNS, astrocytes of the optic nerve head do not express AQP4 [14]. To determine if this lack of AQP4 is correlated with the boundary of solute penetration from CSF into the retina, we injected 70 kDa dextran tracer into the CSF and imaged dextran fluorescence in optic nerve head sections that were additionally stained with antibodies to AQP4 and the vascular endothelial antigen CD31 (Fig. 4E). Dextran was observed in areas of the optic nerve head which did not express AQP4 demonstrating that the absence of AQP4 does not prevent tracer entry. We conclude that transport of solutes from CSF to the optic nerve is not by an AQP4-dependent glymphatic mechanism.

### Optic nerve uptake of antibodies from cerebrospinal fluid

**Fig. 5:**
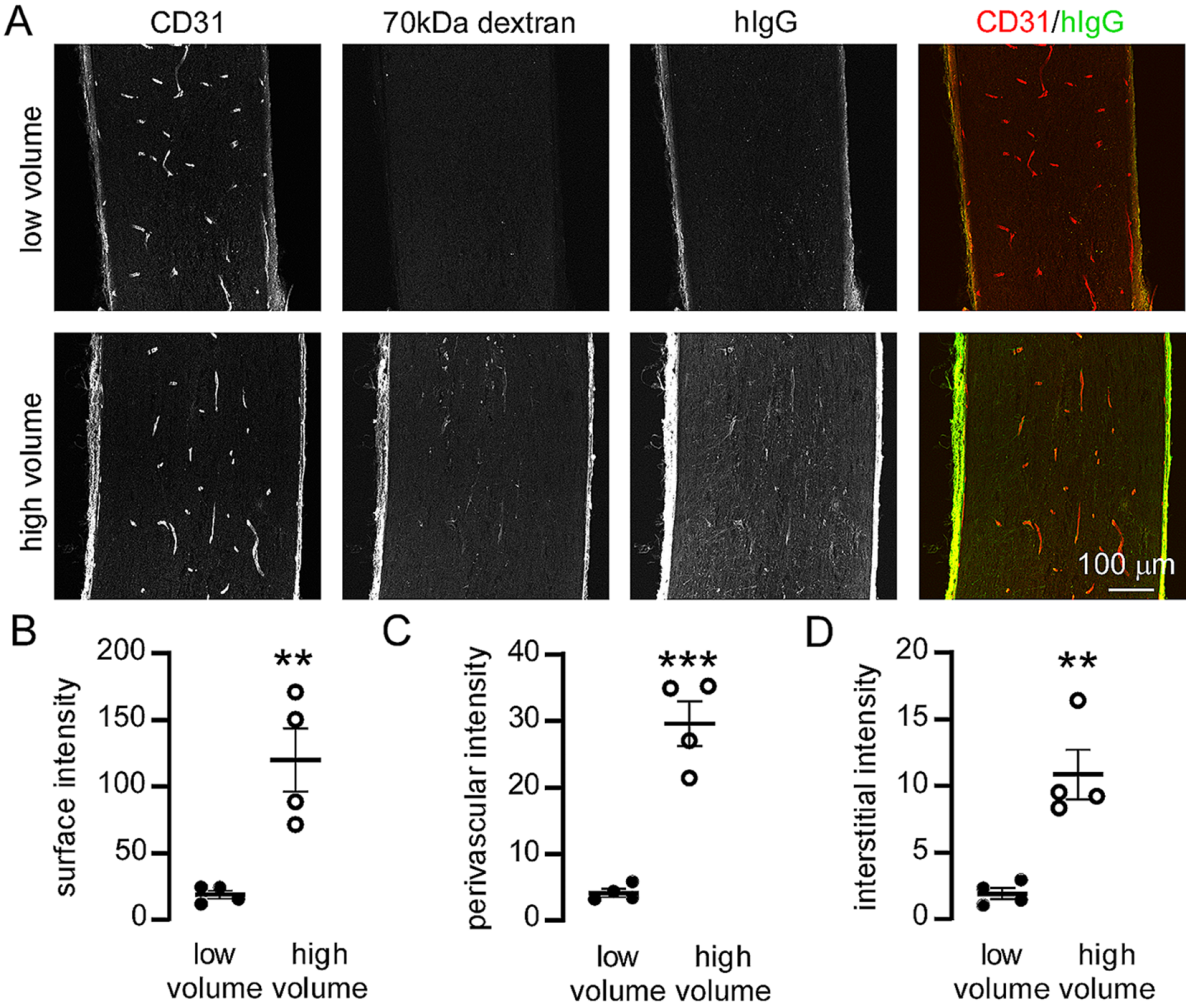
Cisternal injection conditions determine the amount of therapeutic antibody that accumulates in the optic nerve. (**A**) Staining of optic nerve sections with anti-human secondary antibodies reveals uptake of human IgG throughout the optic nerve following high volume cisternal injections of human IgG and 70 kDa dextran tracer. (**B–D**) Quantification of the fluorescence intensity of IgG at the optic nerve surface, perivascular spaces and parenchyma of the optic nerve following high or low volume cisternal injection of a fixed amount of human IgG

Antibody therapeutics have successfully been used to treat AQP4-IgG seropositive NMOSD [23]; intrathecal delivery of human IgG (IVIg) has successfully been used to reduce spinal cord pathology in an animal model of NMOSD [24]. To determine if antibody therapeutics can be delivered from CSF into the optic nerve, we co-injected IVIg with 70 kDa dextran into the cisterna magna, using low or high volume injection protocols. The optic nerve was then fixed, removed, sectioned and stained with anti-human antibodies to reveal the IgG distribution. This distribution essentially mimicked that of the dextran tracer, with greatly increased uptake along the optic nerve following high volume injection (Fig. 5A). Quantification of relative IgG uptake in the optic nerve revealed an approximately 10-fold increase in uptake following high volume injection (Fig. 5B–D). These results demonstrate that therapeutically relevant IgG antibodies can be delivered to the full length of the optic nerve under the experimental injection conditions used here.

**Fig. 6:**
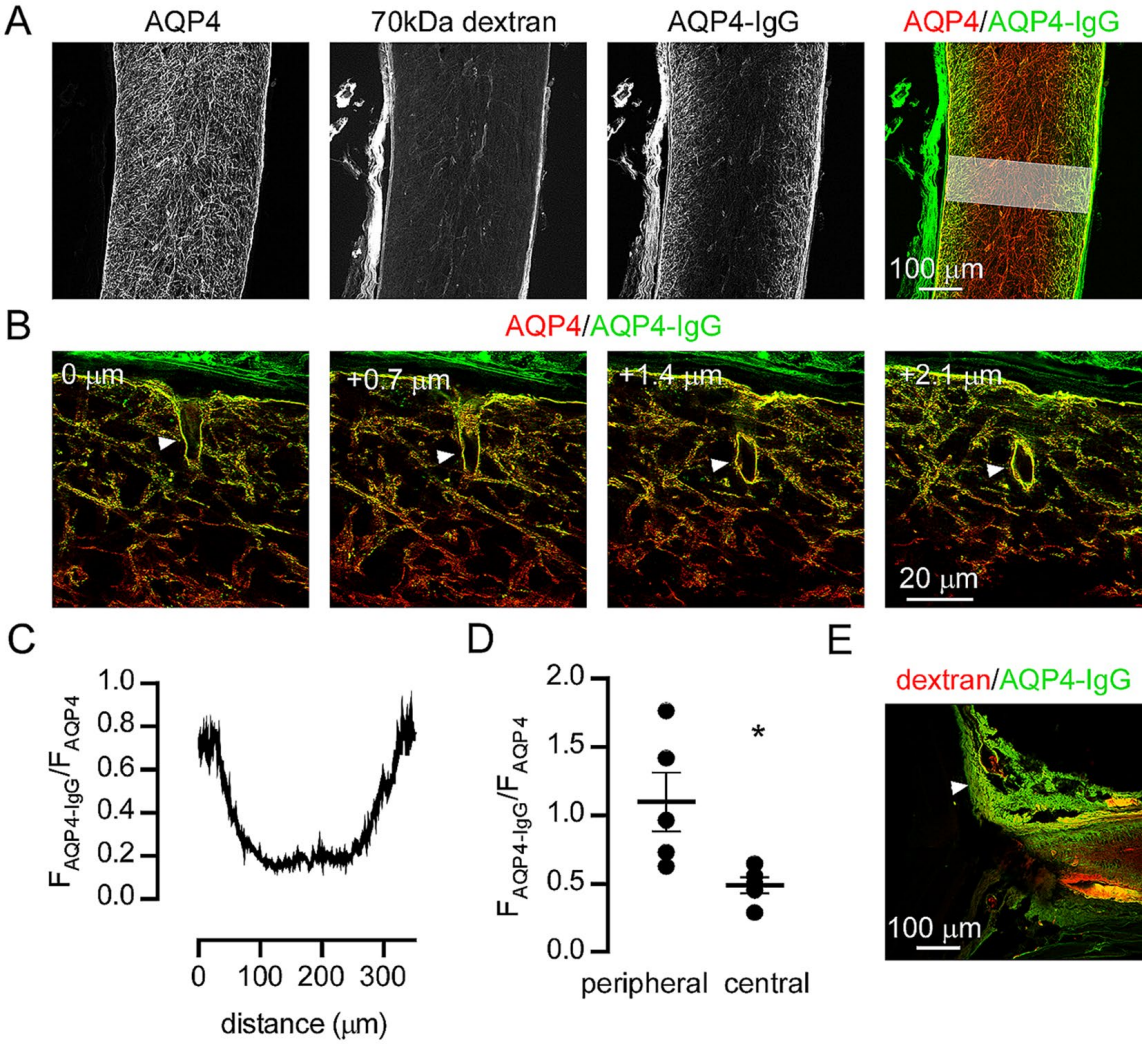
Perivascular and parenchymal uptake of intrathecal antibodies in the optic nerve following cisternal injection. (**A**) Confocal images of optic nerve that was fixed 30 min. after cisternal injection of 70 kDa rhodamine dextran and AQP4-IgG at 5 µl/min, and then subsequently stained with antibodies to AQP4 and with anti-human IgG to detect the injected AQP4-IgG. (**B**) High resolution confocal z-sections showing distribution of AQP4 and AQP4-IgG at the optic nerve surface. Arrowheads demonstrate uptake of AQP4-IgG in perivascular spaces deep within the optic nerve. The relative depth of each optical section is indicated. (**C**) Ratio of AQP4-IgG to AQP4 staining intensity plotted against distance across the optic nerve along the line shown in the right panel of A. (**D**) Ratio of AQP4-IgG to AQP4 in 5 independent experiments measured in peripheral and central areas of optic nerve sections. * p < 0.05 by t-test, n = 5. (**E**) Distribution of injected AQP4-IgG and dextran in a section through the optic nerve head and retina, showing accumulation of AQP4-IgG in the sclera and connective tissues (arrowhead)

Experimental therapeutics derived from high affinity AQP4-IgG clones that have been rendered functionally inactive, can prevent cytotoxicity of AQP4-IgG in vitro and in animal models by competing for AQP4 binding sites [25, 26]. To determine if therapeutics based on recombinant AQP4-IgG could reach the optic nerve following cisternal injection, we injected recombinant, monoclonal AQP4-IgG [18], together with fixable dextran, and measured their distribution in the optic nerve. Following injection, the optic nerve was sectioned and stained with secondary antibodies to human IgG (to detect injected antibody) and with antibodies to AQP4 to determine distribution of AQP4 in the optic nerve. AQP4-IgG was bound to AQP4 throughout superficial regions of the optic nerve but only to perivascular AQP4 deeper in the optic nerve, whereas co-injected dextran was distributed throughout the optic nerve (Fig. 6A). High resolution images of the perivascular space at the surface of the optic nerve showed AQP4-IgG bound to AQP4 along the perivascular space, suggesting that this may be one route for AQP-IgG transport from CSF into the optic nerve (Fig. 6B). However, AQP4-IgG was enriched at the optic nerve periphery (Fig. 6C,D) indicating that additional transport processes such as binding/unbinding reactions or surface diffusion of AQP4/AQP4-IgG complexes, determine AQP4-IgG distribution instead of simple bulk fluid movement. Sections through the optic nerve head demonstrated that AQP4-IgG did not enter the retina and drained into the sclera and connective tissues along with co-injected dextran (Fig. 6E).

## Discussion

**Fig. 7:**
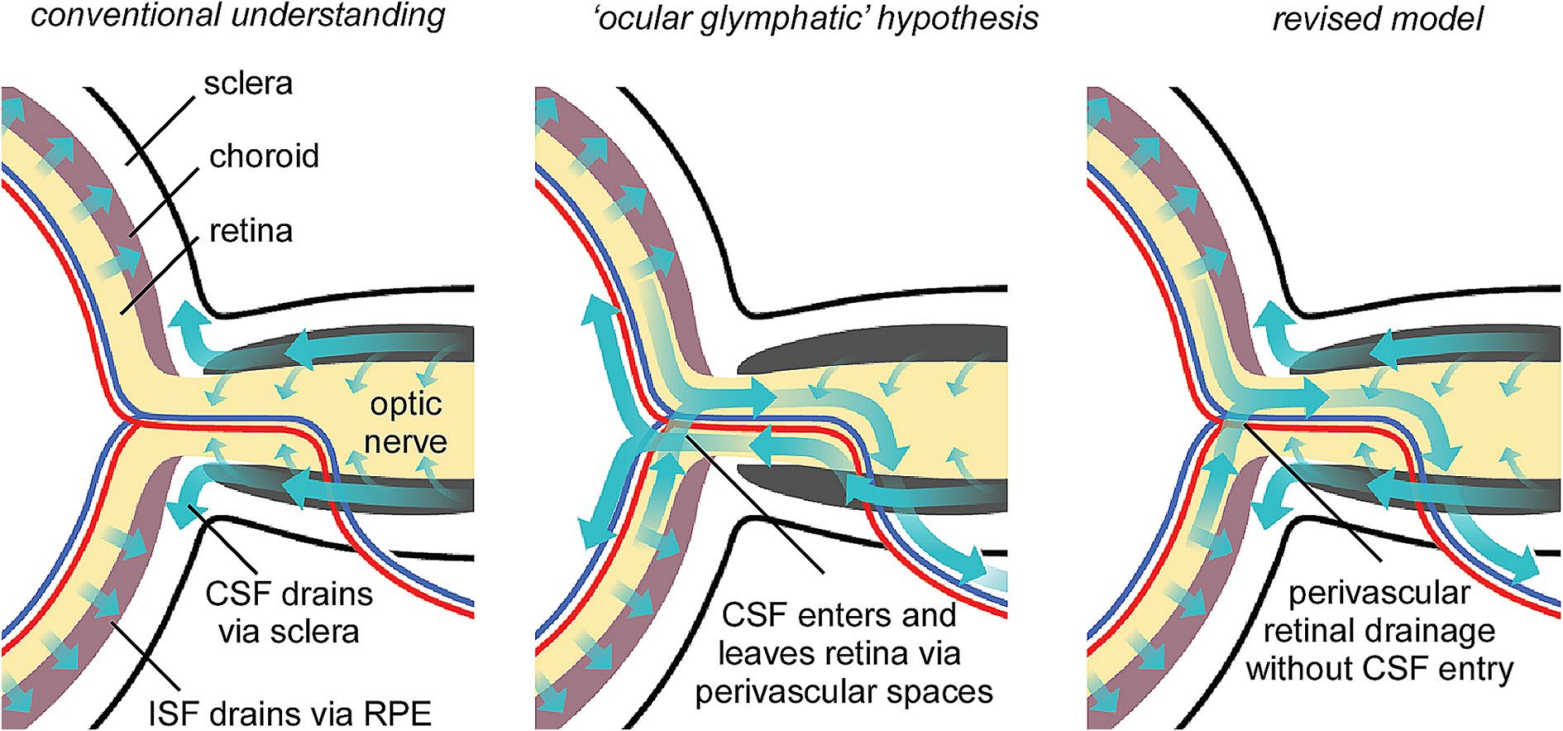
Different models of fluid transport around the optic nerve head. Conventional models (left) propose that CSF drains along the optic nerve sub-arachnoid space to lymphatics in the sclera and connective tissue surrounding the optic nerve. Retinal interstital fluid drains into the choroid via the RPE. The ocular glymphatic hypothesis (center) proposes that CSF circulates through the retina via the central retinal vessels. Based on data presented here, we propose a revised model (right), where CSF does not enter the retina but retinal fluid can drain into the optic nerve head

Here we investigated transport of CSF macromolecules into the optic nerve and retina, and the relevance of this process to the pathology and treatment of NMOSD. We find that macromolecules injected into CSF accumulate in the perivascular spaces and parenchyma of the optic nerve, provided that the injection conditions allow dispersal in the optic nerve sub-arachnoid space. CSF macromolecules do not enter the retina but instead drain via the sclera and connective tissue surrounding the optic nerve head. However, retinal solutes can drain into the optic nerve head via perivascular spaces surrounding the central retinal vessels. These findings, and their relation to previous models of fluid drainage in the optic nerve head and retina, are illustrated in Fig. 7.

Our results confirm previous work demonstrating uptake of CSF solutes in the optic nerve [5]. We further demonstrate that the extent to which CSF solutes accumulate in the optic nerve is highly sensitive to the CSF injection conditions. We have previously shown that high volume injection greatly increases dispersal of solutes within the CSF [16] and that this is associated with increased parenchymal uptake in the brain. Based on these previous results and new findings in optic nerve we propose that the rate of CSF flow along the optic nerve sub-arachnoid space limits the extent to which CSF solutes can enter the optic nerve before being cleared via CSF-draining lymphatics. We also demonstrated that large (2000 kDa solutes are excluded from entering the perivascular spaces of the optic nerve, despite entering the perivascular spaces of the brain under identical injection conditions [16]. Similar findings were reported by Mathieu et al, who showed that solutes over 500 kDa did not enter the optic nerve [5]. Entry of solutes into parenchymal and perivascular regions from the CSF requires transport across the pia via “stomata’ or holes in the pial membrane [27]. Differences in solute size selectivity between brain and optic nerve suggest that there may be differences in the permeability of the pia to solutes between different regions of the CNS. The notion that pial solute permeability is subject to differential regulation is supported by recent findings demonstrating plasticity of pial membrane permeability during aging [2].

We did not find evidence for entry of CSF into the retina, as is proposed by the ocular glymphatic hypothesis [6]. However, our data does support the drainage of fluid from the retina into the optic nerve head via perivascular spaces surrounding the central retinal vessels, in agreement with prior studies [7, 8]. Absence of CSF movement into the retina may simply reflect that drainage to the sclera and connective tissue lymphatics is a lower resistance pathway, rather than any physical barrier to fluid movement between the retina and optic nerve sub-arachnoid space. In support of this hypothesis, ultrastructural analysis has demonstrated pore-like openings in the distal optic nerve sheath that support CSF drainage into the peri-orbital tissue [9, 28]. Drainage via the periorbital pathway may thus represent a significant fraction of the total CSF drainage in mouse [11].

The physiological and pathophysiological importance of the perivascular pathway to fluid drainage from the eye remains to be determined. Perivascular drainage from the eye has been proposed to relieve ocular pressure increase in glaucoma [29], however it seems unlikely that significant quantities of fluid move from the anterior chamber to drain via this pathway [30]. We also found evidence that elevation of intraocular pressure can impair transport of CSF solutes into the optic nerve head. It remains unknown if changes in CSF transport into the optic nerve head caused by elevated IOP contribute to glaucoma pathology. Perivascular transport plays an important role in clearance of interstitial fluid from the brain [1] and may play a similar role in clearance of retinal interstitial fluid [31], however the relative importance of posterior fluid clearance across the retinal pigment epithelium (RPE) and by perivascular routes remains to be determined.

Aquaporin-4 is prominently expressed in perivascular astrocyte endfeet in grey matter and in astrocyte processes throughout white matter [14, 32]. Perivascular AQP4 has been hypothesized to regulate transport of macromolecular solutes between the perivascular space and parenchyma as part of a ‘glymphatic’ system of CNS fluid transport [33, 34]. The existence of a glymphatic system is controversial [13, 35] and it is not known how AQP4, a channel that transports water in response to osmotic gradients, might regulate transport of large molecules such as dextrans and antibodies. It has been proposed that AQP4 regulates transport from the eye to the optic nerve via perivascular spaces as part of the ocular glymphatic system [8], however the absence of AQP4 expression in astrocytes of the optic nerve head [14, 36] demonstrates that AQP4 is not necessary for fluid movement through this area. We did not find any evidence that AQP4 regulates transport of dextrans from the CSF into the optic nerve, however we have not directly tested the possibility that AQP4 regulates fluid transport along the optic nerve, between the optic nerve fibers [8].

A large body of evidence indicates that AQP4-IgG generated by intrathecal B cells plays a key role in the pathology of NMOSD [37]. Distinct AQP4-IgG serotypes are found within the CSF [38], and optic neuritis can be induced in mouse models when AQP4-IgG and complement are repeatedly injected in the cisterna magna [39]. MRI studies have demonstrated that optic nerve lesions in NMOSD are generally restricted to the optic chiasm and adjacent areas, in contrast to MS lesions which are found throughout the optic nerve [40]. Our results regarding the spatial distribution of CSF solutes in optic nerve suggest that the characteristic spatial patterns of NMOSD lesions might be explained because of limited penetration of CSF AQP4-IgG into the optic nerve sub-arachnoid space in the absence of substantial disruption of endogenous CSF movement.

Intrathecal drug delivery has been used to successfully deliver large molecule therapeutics to the brain [41]. Our results here demonstrate that a clinically relevant NMOSD treatment (IVIg) can be delivered at high concentration throughout the length of the optic nerve by injection into the CSF, albeit under the disruptive injection conditions used here. It remains to be determined if therapeutics can be delivered to the optic nerve using less disruptive injection protocols. Additionally, the results demonstrate that AQP4 is not required for optic nerve uptake of CSF macromolecules and therefore that AQP4 loss in NMOSD would not interfere with drug delivery from the CSF. These results, combined with other studies demonstrating efficient spinal cord delivery of NMOSD therapeutics from the CSF [24], suggest that intrathecal delivery of therapeutic antibodies is a potentially useful approach for treating NMOSD cases that are refractory to IV drug delivery.

In summary, this work clarifies aspects of extracellular fluid transport along the optic nerve. We demonstrate that macromolecular solutes from the CSF can access the full length of the optic nerve in an AQP4-independent manner, but that these solutes do not enter the retina and instead drain via the sclera and connective tissue around the optic nerve head. We also find that retinal solutes can drain into the optic nerve, the significance of this process remains to be determined.

## Data Availability

All data and materials are available from the corresponding author upon request.
